# Effects of 6% hydroxyethyl starch 130/0.4 on postoperative blood loss and kidney injury in off-pump coronary arterial bypass grafting: A retrospective study

**DOI:** 10.1097/MD.0000000000006801

**Published:** 2017-05-05

**Authors:** Jeong Jin Min, Hyun Sung Cho, Suyong Jeon, Jong-Hwan Lee, Jeong Jin Lee, Young Tak Lee

**Affiliations:** a Department of Anesthesiology and Pain Medicine; b Department of Thoracic and Cardiovascular Surgery, Cardiac and Vascular Center, Samsung Medical Center, Sungkyunkwan University School of Medicine, Seoul, Republic of Korea.

**Keywords:** acute kidney injury, colloid, hydroxyethyl starch, off-pump coronary arterial bypass grafting, postoperative bleeding

## Abstract

We retrospectively evaluated the effects of 6% hydroxyethyl starch (HES) 130/0.4 on postoperative blood loss and acute kidney injury (AKI) in patients undergoing off-pump coronary artery bypass grafting (OPCAB).

Electronic medical records of 771 patients who underwent OPCAB in our hospital between July 2012 and July 2014 were reviewed, and 249 patients without intraoperative HES-exposure (group NoHES) were matched 1:N with intraoperative HES-exposed 413 patients (group HES) based on propensity score. The effects of intraoperative HES on postoperative cumulative blood loss within the first 24 hours, need for bleeding-related reoperation, and occurrence of postoperative AKI (determined by KDIGO and RIFLE criteria) were analyzed.

In our propensity score matched cohort, there were no significant differences between groups for median postoperative 24 hours blood loss (525 mL in group HES vs. 540 mL in group NoHES, *P* = .203) or need for bleeding-related reoperation (OR, 2.44; 95% confidence interval [CI], 0.64–9.34, *P* = .19). However, postoperative AKI (assessed by 2 criteria) occurred more frequently in group HES than in group NoHES (by KDIGO criteria: 10.7% vs. 3.6%; OR 3.43 [95% CI, 1.67–7.04]; *P* < .001 and by RIFLE criteria: 9.6% vs. 2%; OR 3.32 [95% CI, 1.34–8.24]; *P* = .01). The median volume of infused HES per patient weight was 16 mL/kg in group HES.

In the patients undergoing OPCAB, intraoperative 6% HES 130/0.4 did not increase postoperative bleeding. However, renal safety remains a concern. Intraoperative use of HES should be determined cautiously during OPCAB.

## 1 Introduction

Since its development in the 1960s, intravenous hydroxyethyl starch (HES) solution has been popularly used as a volume expander in hypovolemic patients. However, due to concerns regarding increased risk of mortality and severe renal injury, in July 2013, the US Food and Drug Administration (FDA) recommended avoiding the use of HES in critically ill patients. Whether similar adverse effects occur in the surgical population has not been clarified,^[1]^ and clinicians are advised to avoid HES for patients undergoing open heart surgery with cardiopulmonary bypass due to excess bleeding risk. However, detailed information on the use of different HES products or the use of HES during cardiac surgery without cardiopulmonary bypass is lacking.

The molecular weight and molar substitution of HES have been optimized during the last few decades, and modern starches show better outcomes, especially for coagulation, compared with older starches.^[2–4]^ Recent meta-analyses evaluating the latest generation HES (130/0.4) in surgical patients reported no clear evidence that intraoperative HES increases the risk of adverse outcomes such as mortality or renal injury.^[1,5–7]^ However, the number of clinical trials analyzed was limited, and the majority of included cardiac surgeries were open heart procedures with cardiopulmonary bypass which is known to be associated with hematologic responses and organ injuries.^[8]^

Because off-pump coronary arterial bypass (OPCAB) is a major cardiac surgery that may be accompanied by a large volume of intravenous fluid administration, evaluating the clinical effect of HES on postoperative bleeding and renal injury in this surgical population seems to be essential. Moreover, the effects of HES could be interpreted in OPCAB patients without the complex influences of cardiopulmonary bypass. However, there is insufficient data on the clinical effects of HES in OPCAB.^[2,9,10]^

Therefore, we evaluated the effects of balanced 6% HES 130/0.4 (Volulyte, Fresenius Kabi, Bad Homburg, Germany) on postoperative blood loss and kidney injury in a retrospective propensity score-matched cohort study of patients undergoing OPCAB.

## 2 Methods

### 2.1 Study design and patient population

This study was approved by the Institutional Review Board of Samsung Medical Center (IRB No. 2014-08-058) and was conducted in accordance with the principles of the Declaration of Helsinki. As this was a retrospective study using electronic medical records, the requirement for individual informed consent was waived. The study population consisted of adult patients older than 20 years who underwent OPCAB procedures performed by a single surgeon (Y. T. Lee) between July 2012 and July 2014 at Samsung Medical Center. In June 2013, the US FDA and Health Canada issued a warning on the use of HES solutions; accordingly, the Korean FDA advised health professionals to comply with the same recommendations. Until then, we used 6% HES 130/0.4 in a balanced electrolyte solution (Volulyte, Fresenius Kabi, Bad Homburg, Germany) as volume-expanding fluid in patients undergoing cardiac surgery at our hospital, but have avoided the use of HES solutions during all cardiac surgeries since July 2013. Patients were excluded if they had undergone intraoperative cardiopulmonary bypass or perioperative extracorporeal membrane oxygenation (ECMO) therapy. For patients who underwent several surgeries, we included only the first surgery in the analysis.

### 2.2 Data collection

The electronic medical records of enrolled patients were reviewed, and pre-, intra-, and postoperative data were collected. Data describing postoperative chest tube drainage and laboratory data including blood hemoglobin, platelet, and serum creatinine levels were extracted automatically from the electronic medical records with the aid of the hospital's medical information department. Postoperative outcome data were collected by manual review of each case by 2 researchers (JJM and SJ) who were blinded to the use of HES solution.

### 2.3 Perioperative coagulation management

The perioperative coagulation management strategy was as follows: for all patients who took either aspirin or clopidogrel, the medication was continued until the day of the surgery and resumed as soon as possible after surgery. If the patients had received dual antiplatelet therapy with aspirin and clopidogrel, only aspirin was continued until the day of the surgery, while clopidogrel was discontinued 2 days before surgery. Intraoperatively, the patients were given an initial dose of heparin (1.5 mg/kg) and periodic supplemental doses to maintain an activated clotting time >350 seconds. To neutralize heparin at the end of the surgery, protamine was given in a 0.5:1 ratio to the dose of heparin. If the follow-up activated clotting time was longer than 140 seconds, an additional dose of protamine (10 or 15 mg) was given to further reverse heparin. The perioperative target hemoglobin level was approximately 10 g/dL. The decision on mediastinal re-exploration for surgical hemostasis was made by the attending cardiothoracic surgeons based on the comprehensive monitoring of the postoperative patient's hemodynamic condition and aspects of bleeding (bleeding rate, amount, and/or color of drained blood). The general guidelines for re-exploration in our institution included hourly bleeding rates of approximately more than 400 mL/h for 1 hour; more than 300 mL/h for 2 to 3 hours; or more than 200 mL/h for 4 hours despite continued attempts for coagulopathy correction.

### 2.4 Study endpoints

The primary endpoint was postoperative blood loss measured by cumulative chest tube drainage in the first 24 postoperative hours. Other study endpoints were need for postoperative bleeding-related reoperation and occurrence of postoperative acute kidney injury (AKI). Bleeding-related reoperation was confirmed by manually reviewing hospital records. Postoperative AKI was determined by KDIGO and RIFLE criteria according to creatinine change.^[11,12]^

### 2.5 Statistical analysis

Perioperative characteristics such as patient comorbidities or number of coronary arterial anastomoses might bias clinicians in the choice of intraoperative HES administration. To eliminate this bias, patients receiving HES infusion were matched with those not receiving HES infusion based on propensity score. We used 1:N matching rather than 1:1 matching so as to minimize loss of subjects. Logistic regression was used to calculate exposure propensity scores of likelihood of receiving intraoperative HES using all variables listed in Table 1. As variables for outcome analysis, we included previously identified variables such as age greater than 75 years, body mass index (BMI) lower than 25, reoperation, preoperative low hemoglobin, and other clinical factors as risk factors for perioperative bleeding in cardiac surgery.^[13,14]^ After propensity score matching, the balance between the 2 groups was evaluated using standardized difference, variance ratio, and overall distributions. A measure of standardized difference less than 10% was considered to indicate good balance between groups.

**Table 1:**
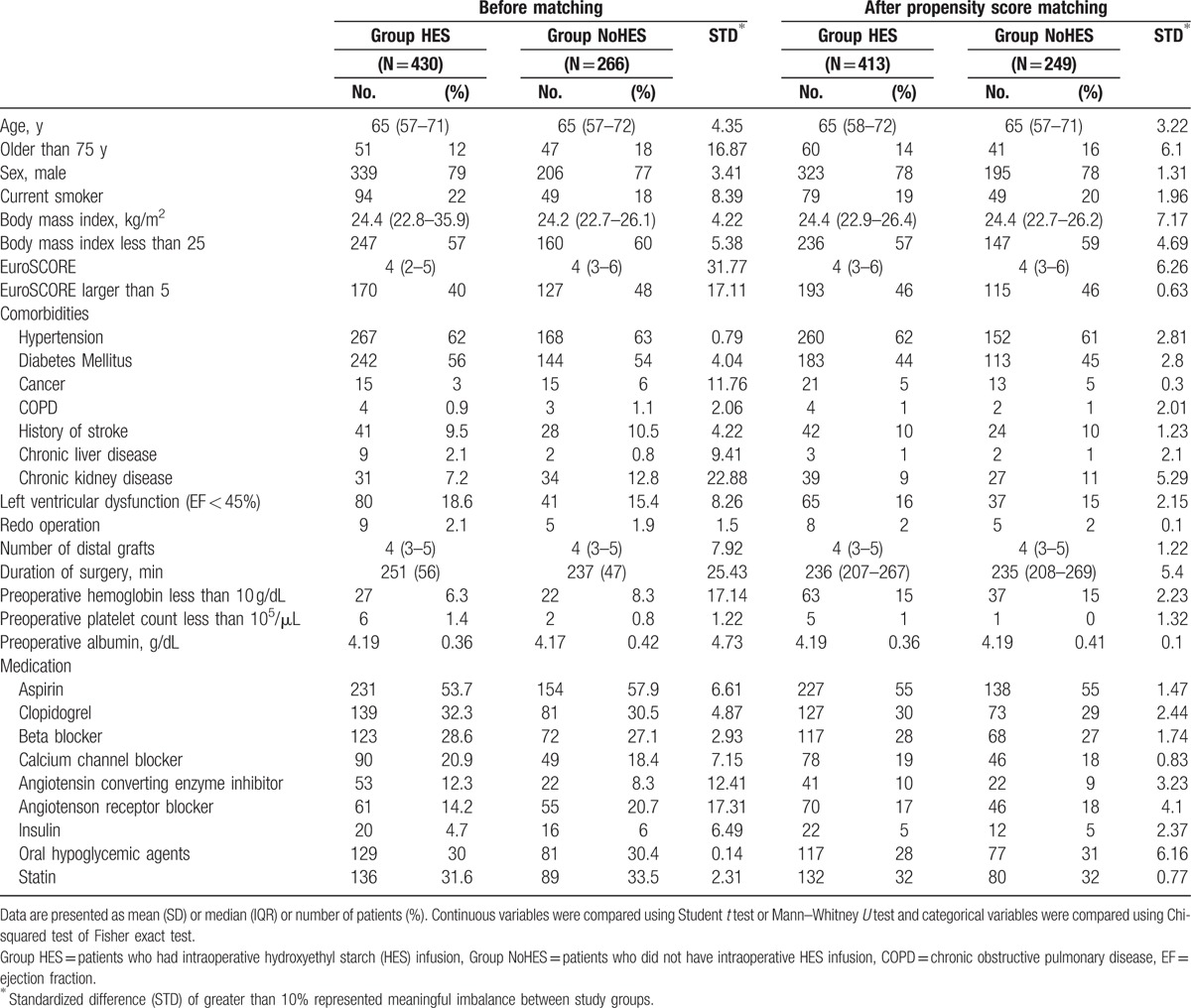
Characteristics of matched variables, before and after propensity score matching.

Continuous variables are presented as the mean (standard deviation [SD]) or median (interquartile range), and categorical variables as numbers and percentages. Normality of the continuous variable was assessed by the Kolmogorov–Smirnov test. To compare patient or surgical characteristics between groups, Student *t* test or the Mann–Whitney *U* test were used for continuous variables, and the Chi-squared test or Fisher exact test were used for categorical variables. For the comparison of continuous outcomes (e.g., postoperative cumulative blood loss, transfused red blood cells) and to estimate the odds ratio (OR) and 95% confidence interval (CI) for risk of dichotomous postoperative outcome according to HES infusion, we used the generalized estimation equations (GEE) method.

To assess the impacts of independent variables on postoperative AKI, univariate, and multivariable logistic regression models were constructed. Variables with a *P* value <.1 in univariate analysis were entered into a multivariable logistic regression model. All statistical analyses were performed using the Statistical Analysis System (release 9.3; SAS Institute, Inc., Cary, NC) or IBM SPSS 22 software (SPSS Inc., Chicago, IL). If *P* < .05, the test was considered to be statistically significant.

## 3 Results

### 3.1 Patient and surgical characteristics

**Figure 1:**
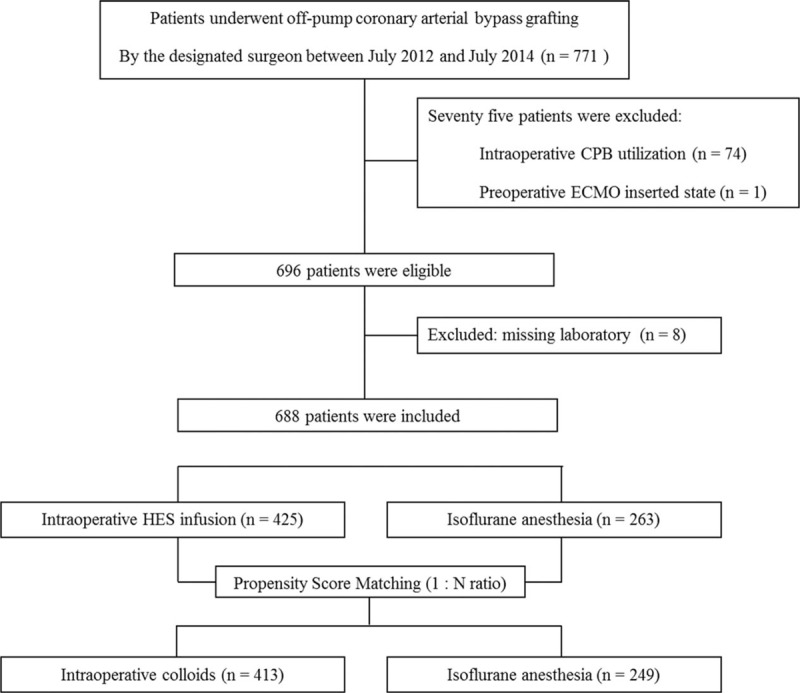
Flow diagram outlining selection of the study population.

Of the 771 patients whose electronic medical records were reviewed, 75 patients who had extracorporeal circulation were excluded (74 had intraoperative cardiopulmonary bypass and 1 had pre- and intraoperative ECMO therapy; Fig. 1). Among the remaining 696 patients, 688 for whom the values of all variables for propensity score matching were available constituted the study cohort. After one-to-many matching according to propensity score, 413 patients who had received intraoperative HES infusion (group HES) were matched with 249 patients who had not (group NoHES), for a total of 662 patients (Fig. 1). Perioperative surgical and clinical characteristics before and after matching are presented in Table 1. The 2 groups had some mismatched variables, including patients older than 75 years, patients with higher EuroSCORE (>5), presence of cancer, chronic kidney disease, or lower preoperative hemoglobin (<10 g/dL), preoperative usage of angiotensin receptor blocker and angiotensin converting enzyme inhibitor, and duration of surgery before propensity score matching (standardized differences >10%). However, there were no significant differences in any variables between groups in the propensity score-matched cohort (Table 1). In the HES group, the median volume of total infused HES was 1000 mL [1000–1500], and the median volume of infused HES according to the patient's body weight was 16 mL/kg [12–21].

### 3.2 Intraoperative HES and postoperative blood loss/bleeding-related reoperation

Postoperative cumulative blood loss was measured by chest tube drainage during the first 24 hours postoperatively. The median amounts of postoperative blood loss in the 2 groups were comparable (median [IQR]: 525 mL [350–760] in group HES vs. 540 mL [400–670] in group NoHES, *P* = .203). In-hospital allogenic packed red blood cell transfusions were also comparable between groups (median [IQR]: 3 unit [2–5] in group HES vs. 3 unit [2–4] in group NoHES, *P* = .243). Of 662 patients, 15 (2.3%) required re-sternotomy for postoperative bleeding. There was no significant difference in the rate of postoperative bleeding-related reoperation between groups (2.9% [12/413) in group HES vs. 1.2% [3/249) in group NoHES, OR 2.44 [95% CI: 0.64–9.34], *P* = .191).

### 3.3 Intraoperative HES and postoperative AKI

The overall incidence of postoperative AKI was 8% (53/662) by the KDIGO criteria and 4.4% (29/662) by the RIFLE criteria. The incidences of postoperative AKI by both criteria were significantly higher in group HES than group NoHES (AKI by KDIGO: 10.7% [44/413] vs. 3.6% [9/249], OR 3.43 [95% CI: 1.67–7.04], *P* < .001; AKI by RIFLE: 5.8% [24/413] vs. 2% [5/249], OR 3.32 [95% CI: 1.34–8.24], *P* = .01, Table 2). In the multivariable logistic regression analysis to identify predictive factors for postoperative AKI, intraoperative use of HES significantly increased the risk of postoperative AKI after adjusting for variables with *P* < .1 in univariate analysis (OR 5.51 [95% CI: 2.22–13.7], *P* < .001 for AKI by KDIGO criteria and OR 6.94 [95% CI: 2.04–23.55], *P* = .002 for AKI by RIFLE criteria, Table 3). Other factors that showed independent association with postoperative AKI were age, presence of preoperative chronic kidney disease, hypertension, redo cardiac operation, and preoperative serum albumin, as shown in Table 3.

**Table 2:**
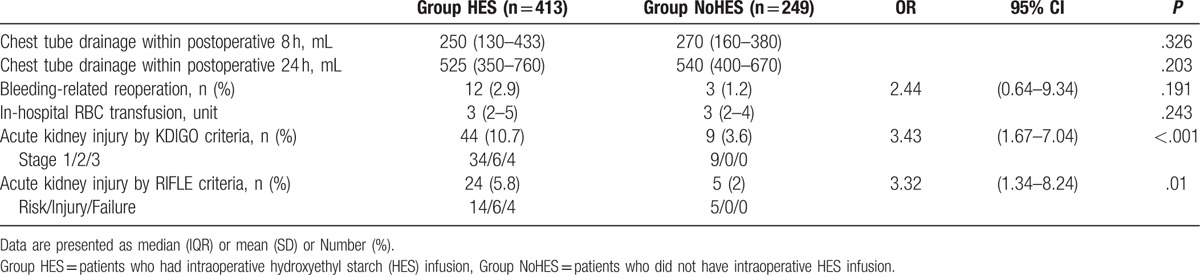
Risks of postoperative complications according to the intraoperative hydroxyethyl starch infusion based on matched data.

**Table 3:**
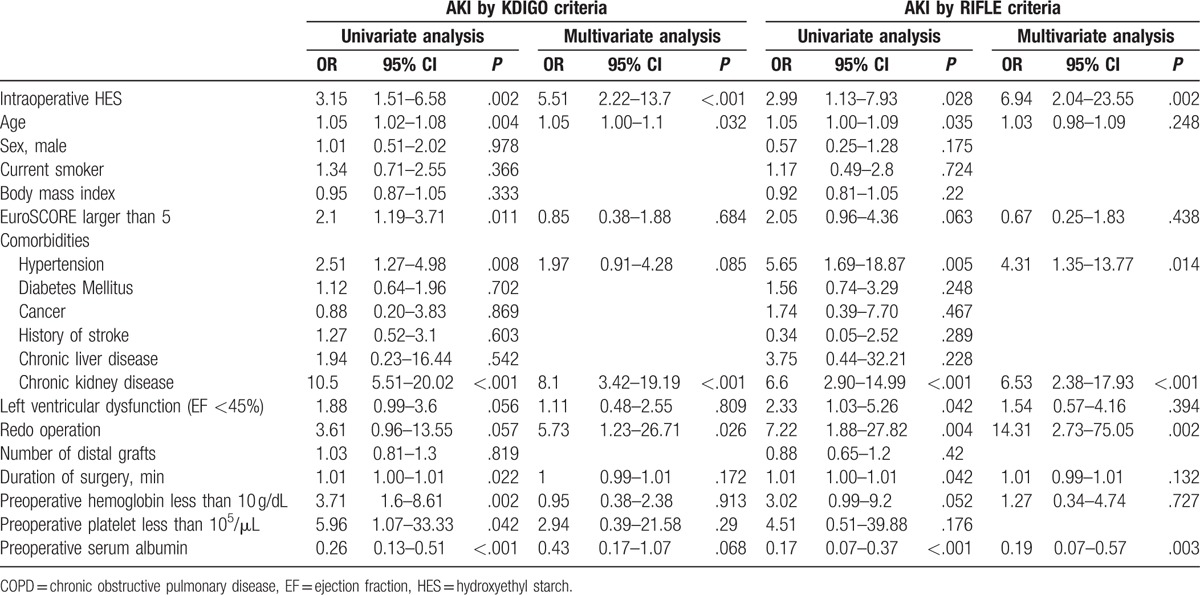
Predictors of postoperative acute kidney injury assessed by the KDIGO and RIFLE criteria.

## 4 Discussion

In this retrospective propensity-score matched study, we evaluated the effects of the intraoperative use of 6% HES 130/0.4 solution on postoperative blood loss and kidney injury in patients undergoing OPCAB. We found that intraoperative HES did not increase the risk of postoperative cumulative blood loss within the first 24 hours or of bleeding related reoperation. However, use of intraoperative HES was associated with occurrence of postoperative AKI.

Intravenous HES solution was previously widely used as a volume expander to treat hypovolemia in surgical patients. However, since the FDA warning against its use in June 2013, coagulation monitoring has been recommended to address concerns regarding excessive bleeding when using HES during on-pump cardiac surgeries. In a previous meta-analysis of cardiac surgeries involving cardiopulmonary bypass, Navickis et al^[15]^ demonstrated that HES increased blood loss and the need for reoperation due to bleeding. Possible mechanisms of colloid-induced coagulopathy were depletion of circulating coagulation factors, interference of large HES molecules with fibrinogen, coagulation factor VIII and von Willebrand factor, delaying kinetics of fibrin, and impairment of platelet function.^[16,17]^ These conditions resulted in a reduction in clot strength and enhancement of fibrinolysis.^[18–20]^ However, those colloid-induced coagulation derangements were based on analyses including older-generation HES with large molecular weights or highly substituted and high C2:C6 ratio, which undergoes slower metabolism.^[15]^ Moreover, in some studies, the clinical relevance of impairments in thromboelastogram tests for assessing actual bleeding has been questionable.^[18,21]^

Although earlier meta-analyses of cardiac surgery reported increased risk of postoperative bleeding in patients who received HES,^[3,15]^ a recent meta-analysis including a larger number of recent randomized trials yielded different results.^[5]^ In a recent meta-analysis of cardiac surgery, Jacob et al^[5]^ included 7 OPCAB studies that were excluded in previous meta-analyses and reported that older starches with mean molecular weights greater than 200 kDa increased blood loss and transfusion requirements, but that these adverse effects were not observed with latest-generation tetrastarches (130/0.4). Such differences in the effects of different generation HES formulations on blood loss or coagulation have also been reported in other previous investigations.^[2,3,22]^ In a meta-analysis, Wilkes et al^[3]^ detected lower postoperative blood loss in albumin versus high MMW HES (450 kDa) but not versus medium MMW HES (200 kDa). In a randomized trial by Muralidhar et al,^[2]^ low-molecular weight HES 130/0.4 led to more rapid postoperative recovery of von Willebrand factor (vWF) and less chest tube drainage than medium-molecular weight HES 200/0.5 or gelatin in OPCAB patients. In an in vitro study using thromboelastometry analysis, Roche et al^[4]^ also found that smaller molecular weight-balanced HES preserves coagulation better than large molecular weight starches.

In the present study of OPCAB patients, the intravenous use of 6% HES 130/0.4 was not associated with early postoperative cumulative blood loss or bleeding-related reoperation. Although fewer in number than studies on on-pump cardiac surgery, several investigations have evaluated the impact of HES on perioperative blood loss and coagulation in OPCAB patients. Earlier OPCAB studies using older-generation HES identified increased risk of bleeding or coagulopathy.^[23–25]^ However, a more recent randomized trial in OPCAB patients demonstrated that the use of 6% HES 130/0.4 up to 30 mL/kg did not increase perioperative blood loss compared with the use of crystalloid.^[9]^

The renal effect of synthetic colloid is another frequently concerned and investigated clinical topic. There is robust evidence of the nephrotoxic effects of colloid solution in critically ill or septic patients.^[26–28]^ The question of whether similar adverse renal effects exist in surgical patients has been investigated in recent reviews and meta-analyses that showed that the use of HES, especially HES 130/0.4, did not increase the risk of postoperative renal dysfunction versus non-starch comparators.^[1,6,7,29]^ However, these meta-analyses mostly included small trials with low event rates, and the authors commented the data insufficiency to identify the renal effects of HES. Moreover, the heterogeneity of the patient samples with various surgical severities included in these trials also raises concerns about the renal safety of HES.

There is only limited data comparing the renal effect of HES compared with crystalloid-only treatment in OPCAB patients. An observational study of 787 OPCAB patients evaluated the effects of perioperative fluid strategy on AKI, but compared different kinds of HES (saline-based versus balanced-solution based), not HES and non-HES.^[10]^ The sample size of another OPCAB study that compared patients treated with HES and crystalloid-only was not calculated to evaluate effects on postoperative AKI, which was a secondary outcome.^[9]^

Previously, the risk of postoperative acute renal failure was reported to be increased in patients undergoing high-risk surgeries or in patients with coronary arterial disease.^[30]^ In this regard, patients undergoing OPCAB surgery may be a more susceptible population for postoperative kidney injury among surgical populations. Moreover, the elimination half-life of HES was variable according to patient comorbidity and severity of surgery as assessed by means such as inflammation and stress levels of surgery.^[31]^ In our study, variable degrees of kidney injuries (KDIGO stages 1–3) occurred more frequently in the HES group even with the infusion of small volumes of tetra-starch under the recommended dose.

Although the exact mechanism of HES-related kidney injury is poorly understood because of insufficient pathologic evidence, the one suggested mechanism of HES-related kidney injury was tissue uptake of HES molecules by luminal epithelial cells in the proximal tubules, even after treatment with modern HES products with lower molecular weight and degree of substitution. This tissue uptake did not seem to be dose- or time-dependent.^[32]^ Another possible mechanism is a hyperoncotic kidney injury in which glomerular filtration rate decreases secondary to a reduction in filtration fraction.^[33]^ Although most kidney injuries were in the mild stage (KDIGO stage 1), there have been reports on the adverse long-term renal effects of reversible mild renal injury after cardiac surgery.^[34]^ Considering that the perfect prediction of postoperative AKI is difficult and that many patients undergoing OPCAB may require critical care after surgery, the intraoperative use of HES should be considered more cautiously until a clear conclusion is made regarding its renal safety. In addition, careful observation of the occurrence of renal injury would be necessary if it was used inevitably according to the patient's condition in OPCAB patients.

There were several limitations in this retrospective study. First, as this was not a prospective randomized study, there could be hidden bias due to confounding factors. Moreover, a prospective randomized trial is the only reliable way to exclude small relative risks. However, it is difficult to randomize patients for cardiac surgery to receive colloids owing to ethical concerns. We tried to adjust as many of the confounding factors as possible that could affect the outcome occurrence. In particular, we matched most previously reported risk factors of postoperative bleeding^[13,14]^ and AKI^[35,36]^ after cardiac surgery between groups. Moreover, the study was conducted in a homogeneous patient sample who underwent OPCAB by a single surgeon and a single anesthesiologist-in-charge. The results of this retrospective study would be worth investigating before large randomized clinical trials in patients undergoing OPCAB. Although we could not adjust for some factors such as the time point at which the operation was performed (more recent population for NoHES group), we think that the 1 to 2 year difference at which the surgery was performed would not significantly alter the study outcomes because the surgeon and anesthesiologist-in-charge, both of whom were experienced, maintained patient management protocol or surgical techniques during the study periods except for the use of HES solution. Second, because the present study sample consisted of all patients who underwent OPCAB during the study period at our hospital, the effect of HES on postoperative blood loss in patients with preoperative coagulopathy cannot be confirmed. Moreover, the mean volume of infused HES in this study was much lower than the manufacturer-recommended limit of 33 mL/kg, and the effects of HES beyond this recommended limit on postoperative bleeding cannot be clarified. Finally, due to the incomplete follow-up data, we were unable to analyze the long-term effects of intraoperative HES on postoperative survival or renal outcomes. As most of the AKI patients in our samples were in mild stages of renal injury, further studies are needed to confirm the long-term effects of HES in OPCAB patients.

In conclusion, in this retrospective study of patients undergoing OPCAB, we found that third-generation HES suspended in balanced-electrolyte solution did not increase the risk of postoperative bleeding when used within the recommended dose. However, there remains concern regarding an association between intraoperative HES and postoperative AKI. Until confirmative results on renal safety are achieved in further studies, the intraoperative use of 6% HES 130/0.4 should be determined very cautiously during OPCAB.
